# Molecular characterization of heavy metal-tolerant bacteria and their potential for bioremediation and plant growth promotion

**DOI:** 10.3389/fmicb.2025.1644466

**Published:** 2025-08-08

**Authors:** Saira Abbas, Sobia Zulfiqar, Muhammad Arshad, Nauman Khalid, Amjad Hussain, Iftikhar Ahmed

**Affiliations:** ^1^National Culture Collection of Pakistan (NCCP), Land Resources Research Institute (LRRI), National Agricultural Research Centre (NARC), Islamabad, Pakistan; ^2^Department of Zoology, University of Science and Technology, Bannu, Pakistan; ^3^Institute of Environmental Sciences and Engineering, School of Civil and Environmental Engineering, National University of Sciences and Technology (NUST), Islamabad, Pakistan; ^4^School of Food and Agriculture, University of Management and Technology, Lahore, Pakistan; ^5^College of Health Sciences, Abu Dhabi University, Abu Dhabi, United Arab Emirates; ^6^Higher Education Commission, Islamabad, Pakistan

**Keywords:** heavy metals, PGPR, tannery discharge, biosorption, bioremediation

## Abstract

**Introduction:**

Heavy metal pollution adversely affects soil health by disrupting the microbial community structure and functions. The current study aimed to isolate and characterize heavy metal-tolerant bacterial strains and evaluate their potential for soil bioremediation and promoting agricultural sustainability.

**Methods:**

A total of 68 bacterial strains were isolated from industrial discharge-contaminated sites and screened for their maximum tolerance limits (MTL) against Cr, Cu, Pb, As, and Cd. The biosorption potential of 23 phylogenetically diverse strains was evaluated. Molecular identification was carried out through 16S rRNA gene sequencing, and plant growth-promoting genes (*acdS* and *nifH*) were screened. Four representative strains (NCCP-650^T^, NCCP-614, NCCP-644, and NCCP-602) were tested for their effect on the growth of *Brassica napus* under axenic conditions with 50 mg/L of each metal.

**Results:**

Several isolates exhibited high MTLs, with tolerance up to 3600 mg/L for Cr, 3300 mg/L for Cu, and 3000 mg/L for Cd and As, while Pb tolerance reached 2100 mg/L. Biosorption was highest for Pb, followed by Cd and Cu; Cr and As were less effectively biosorbed. Molecular identification revealed affiliation of strains to 19 bacterial genera, with *Bacillus* (21%), *Pseudomonas* (12%), and *Staphylococcus* (10%) as dominant. Seven strains harbored both *acdS* and *nifH* genes, with 15 and 8 strains positive for *nifH* and *acdS* individually. In plant experiments, all four tested strains improved *B. napus* growth under heavy metal stress, with NCCP-650^T^ showing the most significant enhancement.

**Discussion:**

The isolated strains demonstrated significant tolerance and biosorption of toxic metals, along with plant growth-promoting potential. These findings suggest that selected isolates, particularly NCCP-650^T^, can serve as bioinoculants for enhancing plant growth and bioremediation in metal-contaminated environments.

## Introduction

Anthropogenic activities introduce multiple pollutants into the environment, consequently increasing the environmental burden (Zafar et al., 2021; Altaf et al., 2021). Heavy metals are continuously released into the atmosphere through industrial processes, causing serious environmental concerns (Arshad et al., 2020; Gul et al., 2021; Manzoor et al., 2021). Some of these heavy metals also contain elements that are vital for living organisms at considerably low concentrations (Alloway, 1990). However, relatively high concentrations of these elements can have a toxic impact on the fauna and flora (Ilyas et al., 2022; Natasha et al., 2022). These elements usually include transition metals in high densities (>5 g cm^−3^) compared to other materials. Soils are regarded as natural resources for producing food and other raw materials for human use. Nevertheless, soil also acts as a sink for waste materials, including heavy metals (Park et al., 2011). Phytoextraction has the potential to restore or manage contaminated soils by providing a cost-effective solution (Arshad et al., 2008; Manzoor et al., 2018, 2019a). However, conventional remediation technologies are less effective and can sometimes impose detrimental effects on soil quality (Biswas et al., 2015; Wan et al., 2016).

Numerous plant species can grow in heavy metal-contaminated soils, but they cannot be used for remediation purposes due to their slow growth, low accumulation potential, and very low biomass (Shen and Liu, 1998). Moreover, heavy metals are occluded or adsorbed by iron–manganese oxides or complexes, organic matter, primary or secondary metabolites, and carbonates (Garbisu and Alkorta, 2001). These metal complexes limit heavy metal bioavailability in the soil and reduce phytoremediation efficiency (Chen et al., 2004; Sheng and Xia, 2006). To improve the phytoavailability of metals, multiple strategies have been adopted, including the use of chelators, bacteria, fungi, and organic and inorganic amendments (Manzoor et al., 2019b; Gul et al., 2019a,b, 2020; Arshad et al., 2016). Several rhizospheric bacteria have been reported to be tolerant or resistant to the toxicity of numerous heavy metals (Cardón et al., 2010; Dary et al., 2010; Koo and Cho, 2009; Tank and Saraf, 2009). Intrinsic microbial properties enable these bacteria to tolerate the toxic effects of heavy metals, while their metal resistance ability is attributed to metal detoxification mechanisms that are activated upon exposure to elevated concentrations of heavy metals (Ledin, 2000).

Bacteria develop various mechanisms to promote plant growth in soils with high concentrations of heavy metals, namely, biosynthesis of 1-aminocyclopropane-1-carboxylic acid (ACC) deaminase, production of phytohormones and siderophores, and production of indole acetic acid (Manzoor et al., 2021). Plant growth-promoting rhizobium (PGPR) strains have shown promising results in laboratories and greenhouse studies; however, the responses observed in field trials are inconsistent (Bowen and Rovira, 1999). PGPRs not only increase the growth of plants but also mediate the remediation of metal-polluted soils in close association with plants (Zhuang et al., 2007). Studies have shown that PGPR strains play crucial roles in metal tolerance and plant growth improvement in metal-contaminated soils (Cardón et al., 2010; Dary et al., 2010; Koo and Cho, 2009; Tank and Saraf, 2009).

Biological nitrogen fixation is important for maintaining the fertility of the soil system, which results from a series of nitrogenase enzymes. These nitrogenase enzymes are complex with heterotetrameric cores and are encoded by the *nifK* and *nifD* genes. Moreover, these enzymes have a dinitrogenase reductase subunit encoded by the *nifH* gene (Gaby and Buckley, 2014), which transfers reducing equivalents to the core enzyme complex and converts nitrogen (N_2_) into ammonia (NH_3_). The *nifH* gene is widely used to study the ecology of nitrogen-fixing bacteria. The diversity of nitrogen-fixing bacteria varies with habitat, and heavy metal-polluted soils could be valuable habitats for studying the complexity of *nifH* genes (Gaby and Buckley, 2014). Soil microbes can potentially affect the bioavailability and mobility of heavy metals in plants. Rhizospheric bacteria can increase the uptake of nickel (Ni) in *Alyssum murale* and cadmium (Cd) in *B. napus* (Abou-Shanab et al., 2006; Sheng and Xia, 2006). Similarly, the heavy metal-tolerant strain of the PGPR, *Bacillus subtilis* “SJ-101”, improved the growth of *Brassica juncea* in the presence of Ni toxicity (Tank and Saraf, 2009). The presence of PGPR strains, *Acinetobacter* and *Pseudomonas,* improved the mobility of a few important metals in plants (Esitken et al., 2006). Arbuscular mycorrhizal fungi also stimulate the phytoextraction process by forming associations with plant roots that enhance the uptake of both natural and toxic heavy metals. They also improve plant growth characteristics and increase total metal accumulation (Wang et al., 2007).

Biosorption is the ability of certain types of microbial biomass to accumulate heavy metals from aqueous solutions. Agricultural wastes efficiently adsorbed copper (Cu), Ni, lead (Pb), Cd, and zinc (Zn). Oves et al. (2013) studied the biosorption potential of *Bacillus thuringiensis* and showed that the strain can biosorb 94% Ni, 91.8% Cu, and 87% Cd.

In this context, the overall objective of the study was to develop a strategy for the bioremediation of heavy metals while ensuring the growth of crop plants. The specific objectives included the following: (1) isolating and characterizing the metal-resistant bacteria from different industrial discharge sites in Pakistan; (2) evaluating the biosorption potential of the isolated strains for Pb, Cd, Cu, chromium (Cr), and arsenic (As); and (3) assessing the growth-promoting ability of potential biosorbent strains in *Brassica napus* plants via greenhouse experiments. In addition to newly isolated strains, this study included previously described novel taxa such as *Acinetobacter pakistanensis* sp. nov. (Abbas et al., 2014), *Alcaligenes pakistanensis* sp. nov. NCCP-650^T^ (Abbas et al., 2015a), and *Bacillus malikii* sp. nov. (Abbas et al., 2015b), which were functionally characterized here for the first time for their biosorption efficiency and plant growth-promoting potential under heavy metal stress.

## Materials and methods

### Sample collection and isolation of heavy metal-tolerant bacteria

Soil, sewage, and/or water samples were collected in sterilized plastic bottles from the discharge waters of the tannery industry areas of Sialkot, Kasur, and Islamabad in Pakistan. The samples were subsequently brought to the laboratory and stored at 4°C until further use. The effluent samples were analyzed for heavy metals (Pb, Cd, Cu, Cr, and As) using an atomic absorption spectrophotometer (Perkin–Elmer, USA). Standard stock solutions (1,000 mg/L) of the metals were procured from Sigma–Aldrich, USA.

For the isolation of bacterial strains, the samples were diluted in phosphate-buffered saline (PBS) solution supplemented with increasing concentrations of heavy metals (100 mg/L/day). The supernatant was streaked on agar plates containing nutrient agar (NA) from different media, tryptic soya agar (TSA), or marine agar (Difco™, USA) supplemented with 600–1,200 mg/L of heavy metals (Pb, Cd, Cu, Cr, and As). The plates were then incubated at 28°C. The heavy metals were added using the salts lead nitrate (Pb(NO_3_)_2_). Cadmium nitrate (Cd(NO_3_)_2_), copper sulphate (CuSO_4_.4H_2_O), potassium dichromate (K_2_Cr_2_O_7_), and sodium dihydrogen arsenate (NaH_2_AsO_4_). Growth was observed after 24–72 h or until the appearance of bacterial colonies. The isolated colonies exhibiting distinct morphologies (in terms of texture, shape, margin, color, and elevation) were further purified via the subculturing method. The purified cultures of the bacterial strains were maintained on agar plates and stored at-80°C in 35% glycerol stock solution.

### Characterization of the isolated bacterial strains

The purified bacterial colonies were morphologically characterized based on colony color, form, elevation, and margin. The cells of the isolates were also analyzed for Gram staining, morphology, and motility using a microscope (Olympus, CX31 equipped with Digital Camera 5A). Growth characteristics of the bacterial strains were determined across a pH range of 4 to 9, a temperature range of 3 to 50°C, and their tolerance to NaCl concentrations of 0–30%. The cells were grown in tryptic soya broth at a range of pH values, and growth was observed after 24 h using a spectrophotometer (IMPLEN, Germany) at a wavelength of 600 nm. The temperature range was determined by growing the cells on TSA and incubating them at various temperatures (4 to 50°C). Salt tolerance was determined by growing bacterial strains on TSA plates supplemented with NaCl at concentrations ranging from 0 to 30%, with 1% increment.

### Screening of bacterial isolates for maximum metal tolerance limits

The maximum tolerance limit (MTL) for each heavy metal by the isolated bacterial strains was determined according to the methods described by Malik and Jaiswal (2000), in which the MTL was defined as the highest concentration at which visible bacterial growth was observed, while complete inhibition of growth at the next higher concentration was considered the threshold. For MTL evaluation, the media were supplemented with various heavy metals (Pb, Cd, Cu, Cr, and As) using their salts as mentioned above, initially at a concentration of 300 mg/L, with a gradual increase of 300 mg/L up to the MTL for the tested isolate. The heavy metal-containing plates were subdivided into four equal sectors, and the isolates were streaked separately in each quarter. The same procedure was carried out with control plates (those plates without metal). Each sample was analyzed in triplicate. Finally, the plates were kept at 28°C for 4 to 6 days to observe the growth of bacteria. The MTL concentration at which the tested isolate failed to grow was subsequently determined.

### Biosorption of heavy metals

To determine the biosorption of heavy metals, 22 strains (which exhibited maximum tolerance against different heavy metals) were tested in this study, with 3 independent replicates. *Escherichia coli* was included as a biological reference strain. Each isolate was cultivated in 5 mL of tryptic soy broth (TSB) supplemented with 50 mg/L of a single heavy metal (Pb, Cd, Cu, Cr, or As) and incubated at 30°C with shaking for 48 h. Bacterial growth was monitored by measuring the optical density at 600 nm (OD₆₀₀) to ensure consistent culture density across all assays. After incubation, 2 mL culture samples were centrifuged at 7,500 × *g* for 10 min. The supernatants were collected to determine the residual metal concentration, while the pellets were dried at 60°C overnight and digested in a 5:3 mixture of nitric acid and perchloric acid. The metal content in both the supernatant and biomass was quantified using inductively coupled plasma–optical emission spectrometry (ICP–OES, Optima 8300, Perkin Elmer, USA). Biosorption was calculated based on the difference between the initial and final metal concentrations. In addition, abiotic controls (metal-containing medium without bacterial inoculation) were included to account for non-biological metal precipitation or adsorption (Chen et al., 2006). Statistical analysis was performed following the GLM procedure in SAS (version 9.4) (SAS Institute, Inc., Cary, USA).

### Identification of the bacterial strains

The bacterial strains were identified based on the sequence analysis of the 16S rRNA gene, as described previously (Ahmed et al., 2007). For this purpose, the DNA template was extracted from fresh cells of the strain by colony PCR at 94°C for 10 min. The 16S rRNA gene was amplified in a thermal cycler (Applied Biosystems, Veriti, USA) by using a Premix Ex-Taq Kit (Takara Cat # RR003A, Japan) with forward primer 9F (5′-GAG TTT GAT CCT GGC TCA G-3′) and reverse primer 1510R (5′-GGC TAC CTT GTT ACG A-3′) under the following PCR conditions: pre-denaturation for 2 min at 94°C (1 cycle), denaturation for 1 min at 94°C (30 cycles), primer annealing for 1 min at 50°C, extension for 1.30 min at 72°C, and a final extension for 5 min at 72°C. The amplified 16S rRNA gene was visualized on a 0.8% (w/v) agarose gel and subsequently purified using a purification kit (Invitrogen, USA), according to the manufacturer’s protocol. The purified PCR product of the 16S rRNA gene was sent for sequencing by Macrogen, Korea,^1^ using the forward primer 27F (5′-AGA GTT TGA TCM TGG CTC AG-3′) and the reverse primer 1492R (5′-ACC TTG TTA CGA CTT-3′). The software package ‘BioEdit’ was used to edit and construct an assembled consensus sequence, which was subsequently subjected to a BLAST search against the DNA Data Bank of Japan (DDBJ) and the EzTaxon server for identification of the strains. For the phylogenetic analysis of the isolated strains, the sequences of the closely related 16S rRNA gene were retrieved from validly published databases. Sequence alignment was carried out by using ClustalW (version 1.6) (Thompson et al., 1997), and phylogenetic analysis was performed to determine the evolutionary relationship of the strain with other validly published strains. Phylogenetic trees were constructed using three algorithms (data not shown in this study): maximum parsimony (MP), maximum likelihood (ML), and neighbor-joining (NJ), which were generated with the software package MEGA-6 (Tamura et al., 2013). The DNA sequences were submitted to the DNA databank of Japan, whose accession numbers are listed in Supplementary Table S1. The strains were submitted to the National Culture Collection of Pakistan (NCCP), and the most important strains (with respect to biosorption capacity or novel species) were also deposited in the Japan Culture of Microorganisms (JCM).

### Screening of bacterial strains for the *nifH* and *acdS* genes

The screening of isolates for the presence of the *nifH* and *acdS* genes was performed using different primer sets to detect the specific amplicon of the respective gene. For this purpose, genomic DNA was extracted from young bacterial cells (14–16 h) using a QIAamp DNA Mini Kit following the manufacturer’s instructions (Qiagen Cat # 51304, Germany).

Amplification of the *acdS* gene was performed in a 50 μL volume using a Premix Ex-Taq kit (Takara Cat # RR003A, Japan) with four sets of primers (Supplementary Table S2) and 50–100 ng of genomic DNA as a template. Amplification was performed with the following PCR procedure: pre-denaturation for 5 min at 94°C (1 cycle), denaturation for 30 s at 94°C (30 cycles), annealing for 30 s at 50°C, extension for 30 s at 72°C, and a final extension for 7 min at 72°C (Blaha et al., 2006). The DNA band of the expected amplicon size was analyzed with each primer set on a 0.8% agarose gel.

Amplification of the *nifH* gene was performed using a Premix Ex-Taq kit (Takara Cat # RR003A, Japan) with three sets of primers (*PolF/PolR, nifHF/nifHI,* and *nifHfor/nifnai*; Supplementary Table S2) and 50–100 ng of genomic DNA as a template using the aforementioned PCR conditions for each set of primers (Supplementary Table S2) (Laguerre et al., 2001; Poly et al., 2001; Sarita et al., 2008). The DNA band sizes of the expected amplicons with each primer set were analyzed on a 0.8% agarose gel.

### Evaluation of heavy metal-tolerant strains for the growth promotion of *Brassica* plants

**Figure 1:**
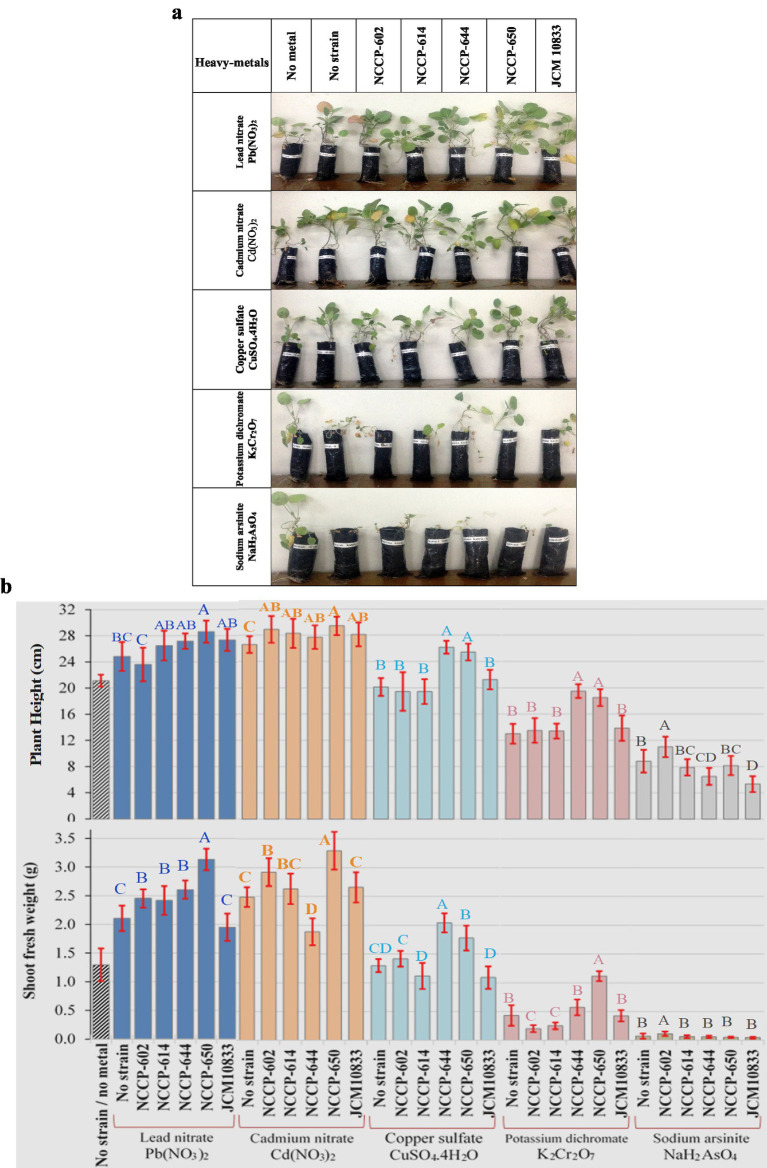
Effect of selected heavy metal-tolerant plant growth-promoting rhizobium (PGPR) strains on the growth of *Brassica napus* under five individual heavy metal stress conditions (Pb, Cd, Cu, Cr, and As at 50 mg/L each): **(a)** Representative images of plants grown under different treatments and **(b)** growth responses of *Brassica napus* to each strain–metal combination. The bars represent the means ± standard errors (*n* = 3). Statistical analysis was performed using the GLM procedure in SAS 9.4. Different letters indicate statistically significant differences among treatments (*p* < 0.05) based on post-hoc comparisons of least squares means.

A greenhouse experiment was performed to test the PGPR activity of selected heavy metal-tolerant strains (NCCP-602, NCCP-614, NCCP-644^T^, and NCCP-650^T^) and a reference nitrogen-fixing strain, *Bradyrhizobium diazoefficiens* JCM 10833, in soil-packed plastic pouches. *Brassica napus* seeds were grown in plastic pouches filled with soil and were watered with 50 mg/kg of each of five heavy metals, namely Pb, Cu, Cr, Cd, and As, separately (using salts Pb(NO_3_)_2_, CuSO_4_.4H_2_O, K_2_Cr_2_O_7_, Cd(NO_3_)_2_, and NaH_2_AsO_4_, respectively) during the whole growth period. The experiment was performed according to a complete random design (CRD) with three independent replications, keeping the strains as a significant factor than those in the heavy-metal treatment. The plants were harvested after 68 days of growth, and plant growth parameters (shoot length and shoot dry weight) were recorded. The statistical analysis was performed following the GLM procedure in SAS version 9.4 (SAS Institute, Inc., Cary, USA). Least squares means were estimated for the main effects of the metal and strain as well as their interaction effect. The standard error of the difference between means was calculated using the estimate statement in the model. In Figure 1, statistical differences among treatment groups are indicated by alphabetic letters, based on post-hoc comparison of least squares means.

## Results

### Physicochemical analysis of the effluent

Effluent samples were analyzed for various physicochemical parameters, including metal ion discharge, pH of the effluent samples, and physical appearance. The pH of the samples was mostly alkaline (pH 7.5 to 8.5), while the majority of these samples were highly colored and had a foul smell. The Cr and Pb concentrations in the majority of the samples were above the permissible limits of 0.1 mg/L.

### Isolation and morphological characterization of bacterial strains

A total of 68 strains were isolated from the discharge of industrial areas (Islamabad, Sialkot, and Kasur) in Pakistan, and the purified strains were subsequently designated as NCCP-601 and onward. The isolated strains were enriched with different heavy metals and differentiated based on colony morphology (Supplementary Table S1). The majority of the strains were round, lobed, and filamentous in shape, with entire margins; however, some strains had irregular margins. The colony colors of the majority of strains were white and pale yellow, while some strains were peach and off-white. The morphologically different strains were further subjected to other experiments and stored in a freezer at −80°C.

### MTL of isolated bacterial strains for heavy metals and NaCl

**Figure 2:**
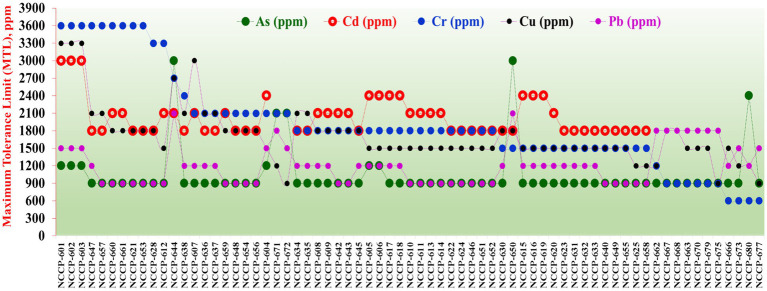
Maximum tolerance limits (MTLs) of isolated bacterial strains for different heavy metals (Cr, Cu, Pb, Cd, and As).

All the enriched bacterial strains were found to be highly tolerant to different heavy metals, including Cr, Cu, Cd, Pb, and As (Figure 2). The results demonstrated that the majority of the isolated strains tolerated 3,600 mg/L Cr, 3,300 mg/L Cu, 3,000 mg/L Cd, 1,500 mg/L Pb, and 1,200 mg/L As. Among these isolates, NCCP-601, 602, 603, 621, 627, 647, 653, 657, 660, and 661 had maximum tolerance limits (MTLs) for Cr (3,600 mg/L), Cd (2,400–3,000 mg/L), Cu (2,100–3,300 mg/L), Pb (1,200–1,500 mg/L) and As (900–1,200 mg/L). The isolates reported in these studies showed the highest tolerance to Cd and As compared to previous reports. The majority of the bacterial isolates were also found to grow over a wide range of sodium chloride (NaCl) concentrations, ranging from 0 to 20%, as shown in Supplementary Table S1.

### Biosorption of heavy metals

**Figure 3:**
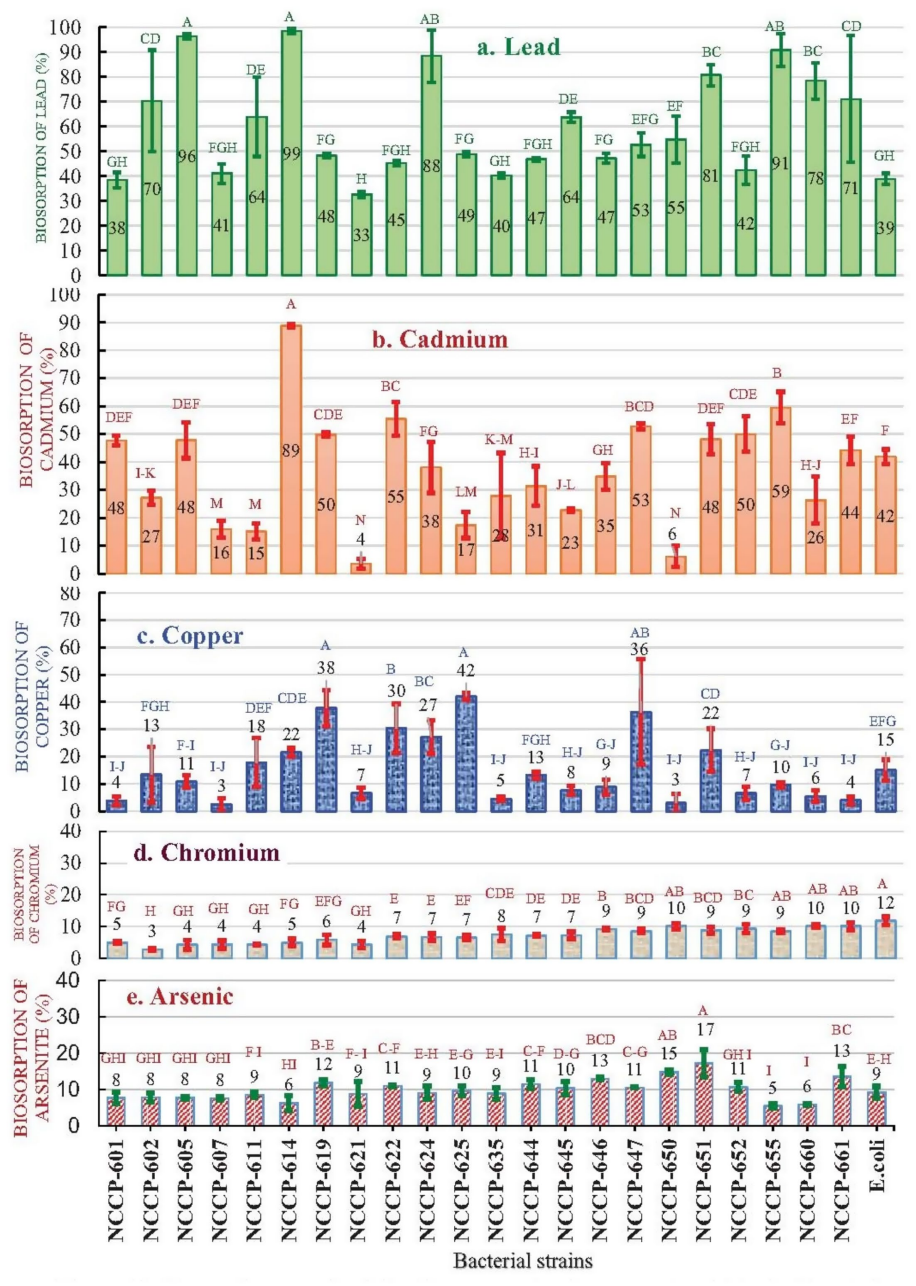
Biosorption capacity (%) of heavy-metal-tolerant strains: **(a)** lead, **(b)** cadmium, **(c)** copper, **(d)** chromium, and **(e)** arsenic. Bars with the same letter for each metal are not significantly different (*p = 0.05*).

Based on the MTL, the isolated strains were further analyzed for possible use in heavy metal biosorption from 50 mL of TSB containing 50 mg/L of the corresponding heavy metals. All the isolated bacterial strains showed significant reductions in heavy metal concentrations in the TSB medium. The maximum biosorption occurred for Pb, followed by Cd and Cu. However, no significant reduction in Cr or As concentration was observed for any of the isolated strains (Figures 3d,e). The isolates significantly differed in their biosorption of Pb, Cd, and Cu (Figures 2a–c), but there was no significant difference in the biosorption of As or Cr (Figures 3d,e). The same alphabetic letter is used for each metal treatment, and the bars in Figure 3 indicate statistically non-significant differences (*p* = 0.05). Among these, three isolates, namely NCCP-614 (99%), NCCP-605 (96%), and NCCP-655 (91%), exhibited maximum biosorption of Pb (Figure 3a). Similarly, strains NCCP-614 and NCCP-655 also exhibited maximum biosorption capacities of 89 and 59% for Cd, respectively (Figure 3b); however, the maximum biosorption of Cu was achieved by NCCP-625 (42%), followed by NCCP-619 (38%) and NCCP-647 (36%) (Figure 3c). Our results indicated that these isolates can be used for the bioremediation of soil and water contaminated with heavy metals such as Pb, Cd, and Cu.

### Identification of the bacterial strains

**Figure 4:**
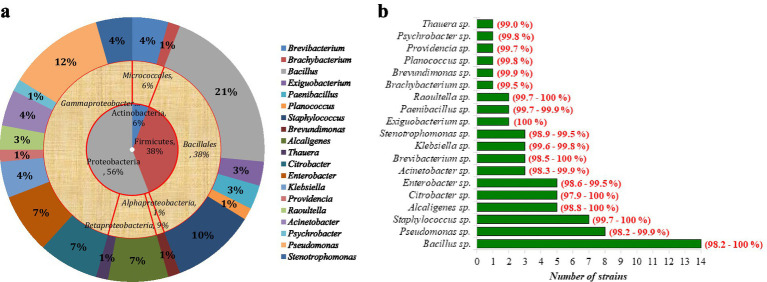
**(a)** Biodiversity pie chart of isolated heavy metal-tolerant strains. The outer ring shows the percentage of the isolated population in a particular genus (see the legend). **(b)** Biodiversity of isolated heavy metal-tolerant strains. The values in front of each bar show the percent similarity range of the strains with the known closely related species of the respective genera.

It is accepted that culturable microorganisms from any given sample taken from an environment represent only a small portion of the total population that is present. In this study, 68 bacterial strains were identified taxonomically based on the sequence of the 16S rRNA gene (Supplementary Table S1). These 68 strains were isolated from effluent samples and found to be highly tolerant to heavy metals. Based on comparative 16S rRNA gene sequence data, a diverse bacterial community was observed (Figures 4a,b). The isolated population (Figure 4a) belonged to three phyla, *Actinobacteria* (6%), *Firmicutes* (38%), and *Proteobacteria* (56%). These heavy metal-tolerant strains (Figures 4a,b) were related to 19 different genera. The dominant strains were *Bacillus* (21%), *Pseudomonas* (12%), and *Staphylococcus* (10%).

The percent 16S rRNA gene sequence similarity with those of closely related species in their respective genera was 97.9 to 100% for *Bacillus*, *Alcaligenes, Acinetobacter, Pseudomonas, Citrobacter,* and *Bravibacterium* (Figure 4b; Supplementary Table S1), which indicates that some of these strains could be characterized taxonomically to delineate them as novel species. Taking these results into account, along with phylogenetic analyses, DNA–DNA homology, and phenotypic and chemotaxonomic data, three isolated strains have been characterized as novel species: *Acinetobacter pakistanensis* sp. nov. (Abbas et al., 2014), *Alcaligenes pakistanensis* sp. nov. (Abbas et al., 2015a), and *Bacillus malikii* sp. nov. (Abbas et al., 2015b). However, the 16S rRNA gene sequences of the other strains had high similarity (>99%) with those of the closely related taxa in their respective clusters; therefore, these strains were not included in the taxonomic characterization studies.

### Screening of the *nifH* and *acdS* genes of isolated bacterial strains

**Figure 5:**
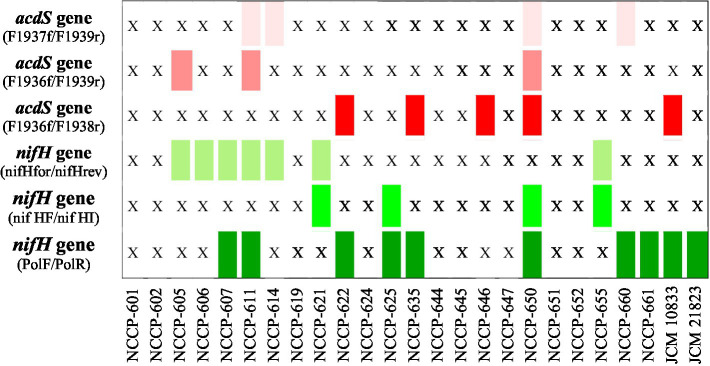
Summary of the amplified PCR products with different markers of the *nifH* and *acdS* genes. Green denotes amplification of the *nifH* gene, and red denotes amplification of the *acdS* gene, while X indicates that no amplification occurred.

To determine the potential use of isolated heavy metal-tolerant isolates in agriculture, phylogenetically different isolates were also analyzed for the screening of the nitrogen fixation gene *nifH* using different primer sets, such as PolF/PolR, nifHF/nifHI, and nifHfor/nifHrev (Supplementary Table S2). *Rhizobium etli* JCM 21823^T^ and *Bradyrhizobium japonicum* JCM 10833^T^ were used as positive controls. The *nifH* gene was amplified by these primers, and amplicons of approximately 360, 420, and 780 bp, previously reported to be associated with the presence of the *nifH* gene, were identified (Supplementary Table S3). The *nifH* gene was amplified in at least 15 isolates with one or two primer sets (Figure 5). Similarly, the presence of the *acdS* gene in the genome was analyzed using four sets of primers, F1936f/F1938r, F1936f/F1939r, and F1937f/F1939r (Supplementary Table S2). The ACC deaminase gene *acdS* was amplified in at least 8 strains by these primers, with approximate sizes of 792, 558, and 516 bp. A careful analysis of the results showed that at least 7 strains (NCCP-650^T^, NCCP-611, NCCP-660, NCCP-635, NCCP-622, NCCP-614, and NCCP-605) were found to have both *nifH* and *acdS* genes (Figure 5). Among them, strain NCCP-650ᵀ, previously described as a novel species (*Alcaligenes pakistanensis*), exhibited the most pronounced plant growth-promoting effect and carried the *nirK* gene (Abbas et al., 2015a), potentially contributing to nitrogen cycling and environmental adaptation. The cooccurrence of functional marker genes with observed biosorption and PGPR traits suggested a possible role for these genes in the adaptive performance of the strains. Overall, the identified strains, particularly those with combined biosorption capacity, PGPR traits, and genetic potential, represent strong candidates for microbial-assisted bioremediation and development as bioinoculants in metal-contaminated agroecosystems.

### Evaluation of heavy metal-tolerant strains for the growth promotion of *Brassica* plants

Based on the presence and absence of the *nifH* and *acdS* genes, as well as MTL, four isolates (NCCP-650^T^, NCCP-614, NCCP-644, and NCCP-602) and the reference nitrogen-fixing strain JCM 10833 were evaluated for their impact on *Brassica napus* growth under axenic conditions when treated with a 50 mg/L solution for each studied metal separately. The statistical analysis revealed that the effects of strain, heavy metals, and their interaction were significant (*p* < 0.05) on shoot fresh weight and length. Additionally, all the strains used in this study played a significant role in increasing the fresh shoot weight and length of *Brassica* plants compared to those of the control plants (with no addition of strain or metal) when irrigated with water containing 50 mg/L Pb or Cd (Figures 1a,b). However, the application of water containing Cr or As salts significantly reduced plant growth, and no positive response was observed in plants irrigated with water containing 50 mg/L As because shoot length and shoot fresh weight were negligible in comparison to those in the other heavy metal treatments. Among the strains tested, NCCP-650^T^ demonstrated the greatest improvement in *Brassica* growth. Additionally, many strains of this genus have been reported to have PGPR activity (Duca et al., 2014).

## Discussion

The biodiversity of heavy metal-resistant bacterial communities from industrial discharge was examined using standard techniques. The initial chemical analysis indicated a slightly high pH level with an ample amount of heavy metal pollution. The discharge sample values exceeded the threshold values described by various environmental protection agencies. Nevertheless, polluted samples contain various bacterial communities that tend to biosorb various heavy metals. Primary characterization revealed different bacterial communities with various shapes and colonies. The composition of waste helps bacterial cells cope with and adapt to external conditions (Mitchell and Kogure, 2006). In this study, we found different bacterial communities based on the composition of industrial waste. The analysis revealed 80 different strains based on phenotypic characterization. However, phenotypic characterization data cannot be used for direct comparisons of different strains (Fritze, 2002).

Molecular characterization was performed with 16S rRNA sequencing, which confirmed the identity of each strain. In our study, we observed the dominant genera *Bacillus,* followed by *Pseudomonas*, *Staphylococcus*, *Alcaligenes*, *Citrobacter,* and *Enterobacter.* Bestawy et al. (2013) identified *Enterobacter*, *Stenotrophomonas, Providencia, Comamonas, Delftia*, and *Ochrobactrum* as dominant genera from activated industrial effluent sludge in Egypt. Gram-positive and gram-negative bacteria have very strong anionic cell walls. This anionic cell wall allows bacteria to bind the metal for nucleation (Kelly et al., 2004). The isolated strains showed good MTL against Cr, Cu, Cd, Pb, and As. The tolerance limit of bacteria against a particular metal gradually increases over time, and bacterial generations produced after a certain time show good resistance against metal concentrations and can be used for decontamination purposes (Kelly et al., 2003). The MTL follows the order of Cr > Cu > Cd > Pb > As; however, the MTL values always vary in relation to the strain type and evaluation parameters during the study.

Heavy metal-resistant bacteria can play crucial roles in the bioremediation of contaminated soil by conferring resistance to heavy metal stresses and reducing toxicity in contaminated soil surroundings (Filali et al., 2000). Microbial ecological studies on mining-impacted sites, such as coal mine dumps, have shown that the structure, diversity, and functional groups of the microbial community can serve as valuable bioindicators of restoration progress and environmental health (Chen et al., 2020). Recent advances in microbial technologies have demonstrated their successful application in the ecological remediation of mine-contaminated soils, often involving plant–microbe partnerships to enhance contaminant removal and ecosystem recovery (Xiao et al., 2021; Guo et al., 2021). In recent years, biosorption has been reported to be a safe and cost-effective process for removing heavy metals from different solutions. The major advantage of biosorption over conventional methods is the efficient removal of heavy metals from different media. The effluents are on the order of only a few parts per billion (ppb) of residual metals (Volesky, 1999). Previous studies have shown that *Pseudomonas aeruginosa* can biosorb both Cr(III) and Cr(VI), with Cr(VI) removal occurring through abiotic reduction to Cr(III), followed by adsorption onto surface functional groups. Amines and carboxyl groups were identified as key binding sites, with protonated amines interacting electrostatically with negatively charged chromate ions under acidic conditions. These findings highlight the role of metal speciation and surface charge in Cr biosorption and may explain the limited Cr uptake observed in strains lacking such reduction capabilities or surface group interactions under the tested conditions (Kang et al., 2007). The genetic makeup and long-term exposure of bacterial strains to these heavy metals could lead to the development of resistance against potential toxicity and adverse effects (Lim and Aris, 2014). Previous studies noted that *S. capitis* could tolerate Cr^+4^ (2,800 μg/mL) and *Bacillus* sp. JDM-2-1 could tolerate Cr^+6^ (4,800 μg/mL). Similarly, these strains were able to resist Cu^+2^ (200 μg/mL), Cd^+2^ (50 μg/mL), Hg^+2^ (50 μg/mL), Pb^+2^ (800 μg/mL), and Ni^+2^ (4,000 μg/mL) (Zahoor and Rehman, 2009). In another study, *Pseudomonas aeruginosa* tolerated Pb^+2^ (650 μg/mL), Cu^+2^ (200 μg/mL), Cd^+2^ (50 μg/mL), Zn^+2^ (50 μg/mL), Ni^+2^ (550 μg/mL), and Cr^+6^ (100 μg/mL) (Rehman et al., 2008). Previous studies have reported that these bacterial isolates exhibit high resistance to heavy metals (Roane et al., 2001).

The ability of isolated microbial populations to tolerate toxic metal concentrations could have been attained by adaptation—a genetically altered tolerance—or by a shift in species composition, whereby organisms that are already tolerant become relatively more competitive (Li et al., 2006). Previous studies have reported the distinctive characteristics of a few members of this genus for antibiotic resistance and Cd reduction (Chien et al., 2007). Regarding Cd reduction with *Stenotrophomonas* sp., our results were well supported by those of a previous study by Chien et al. (2007), which indicated that *Stenotrophomonas* sp. screened from metal-contaminated soil exhibited considerably greater tolerance against heavy metals compared to those acquired from culture collections. A previous study reported that *Staphylococcus* sp. can biosorb Cu^+2^, but its efficiency is directly proportional to the concentration of Cu in the medium (Andreazza et al., 2011; Stanley and Ogden, 2003).

Heavy metal-resistant bacteria of different genera, namely, *Mycobacterium*, *Pseudomonas, Agrobacterium, Achromobacter*, *Arthrobacter*, *Sphingomonas, and Microbacterium,* have been observed to potentially stimulate plant growth and ameliorate stress symptoms in plants (Abou-Shanab et al., 2007; Jiang et al., 2008; Ma et al., 2009). Some rhizobacteria can reduce metal toxicity, resulting in the stimulation of plant growth. In our study, the selected strains increased *Brassica* growth in heavy metal-contaminated soil. These results are promising for Pb, Cd, Cr, and Cu. These results are in agreement with the previous study conducted by Belimov et al. (2005). They isolated and characterized Cd-tolerant bacteria associated with the roots of the metal-accumulating plant *B. juncea* L. Czern. The plants were subsequently grown in metal-polluted soils, during which PGPR strains were selected for their ability to promote plant growth under unfavorable environmental conditions. In support of this finding, Wang et al. (2023) demonstrated that a phosphate-solubilizing fungus combined with native plants significantly reduced soil Pb while enhancing plant biomass and nutrient uptake in a phosphate mining wasteland, reinforcing the potential of plant–microbe systems for heavy metal remediation. He et al. (2009) characterized Cd-resistant bacteria and investigated their potential to promote plant growth. The authors found that Pb and Cd uptake in Cd hyperaccumulator tomato plants cultivated in metal-polluted soil enhanced the phytoremediation efficiency of Cd-polluted soils. In contrast, the isolated strains did not promote the growth of plants in As-contaminated soil since As is considered highly toxic to plants and microorganisms. The reduced growth could be due to the low production of siderophores, which in turn restricted the movement of As in the soil. The production of siderophores is necessary for the mobilization of As in the soil along with iron ions, which renders As more soluble and bioavailable to plants (Drewniak et al., 2008; Wang et al., 2011). Previous studies reported that *Bacillus subtilis* and *Paenibacillus macerans* were able to remove up to 82.2 and 62.4% of As(III), respectively, from an initial concentration of 50 μg/mL. The majority of arsenic removal occurred through surface binding, contributing approximately 90% to *B. subtilis* and 82% to *P. macerans*, while intracellular uptake accounted for a minor portion of the total arsenic removal. The maximum arsenic removal was observed at pH 8, and the optimal temperatures were 35–40°C for *B. subtilis* and 30°C for *P. macerans*. Arsenic binding followed pseudo-second-order kinetics and fit the Langmuir isotherm model. Fourier transform–infrared (FT–IR) analysis indicated the involvement of lipids, carbohydrates, amines, amides, and aromatic groups in the binding process (Vishnoi et al., 2014).

Overall, the diverse metal resistance profiles, biosorption capacities, and plant growth-promoting effects of the isolated bacterial strains underscore their potential for bioremediation and sustainable management of heavy metal-contaminated environments.

## Conclusion

There has been limited research on the biosorption of heavy metals by heavy metal-tolerant bacterial strains. The strains isolated from Pakistan in our studies were found to be highly tolerant to Cr, Cd, Cu, Pb, and As. Some strains were found to be more tolerant to Cd and As than those in previous reports. Our results indicated that two isolates (NCCP-614 and NCCP-655) exhibited more than 91% biosorption of Pb and more than 59% biosorption of Cd, while three other isolates (NCCP-625, NCCP-619, and NCCP-647) exhibited more than 36% biosorption of Cu. These isolates can be used for the bioremediation of soil/water systems contaminated with Pb, Cd, and Cu. Phylogenetic identification of these heavy metal-tolerant strains based on the sequence data of the 16S rRNA gene showed that at least three strains were novel species that can be characterized by polyphasic taxonomy. Molecular characterization of the *nifH* and *acdS* genes revealed that seven strains contained both of these genes. These gene-positive strains also exhibited strong biosorption capacities, suggesting that these genetic traits could contribute to their functional performance in contaminated environments. Among them, the strain NCCP-650ᵀ, which harbored both genes, significantly enhanced the growth of *Brassica napus* in greenhouse experiments under heavy metal stress. This strain has also been reported to possess the nitrite reductase gene (*nirK*), which plays a role in the denitrification process. While this association is promising, further confirmation through gene expression studies and enzymatic activity assays is recommended in future studies. These heavy metal-tolerant strains may serve as potential bioinoculants to improve crop productivity under contaminated conditions. Moreover, the novel taxa identified in this study could be valuable sources of new genes involved in metal transport and tolerance mechanisms. The combined bioremediation and PGPR potential of these strains under stress conditions holds significance for sustainable agriculture.

## Data Availability

The 16S rRNA gene sequencing data presented in the study are publicly available in DNA Data Bank of Japan (DDBJ). The accession numbers associated with the data are given in Supplementary Table S1.

## Footnotes

^1^ http://dna.macrogen.com/eng/
