# Supplementary material for: Molecular characterization of heavy metal-tolerant bacteria and their potential for bioremediation and plant growth promotion
Source: Front Microbiol. 2025 Aug 8;16:1644466. doi: 10.3389/fmicb.2025.1644466 (PMC12370754; doi:10.3389/fmicb.2025.1644466)
Supplement: Supplementary file 1 [file Supplementary_file_1.docx]

**Supplementary Material**

**Molecular characterization of heavy metal tolerant bacteria and their potential in bioremediation and plant growth promotion**

Saira Abbas^1, 2^, Sobia Zulfiqar^1^, Muhammad Arshad^3^, Nauman Khalid^4,5^, Amjad Hussain^5^, Iftikhar Ahmed^1,*^

^1^ National Culture Collection of Pakistan (NCCP), Land Resources Research Institute (LRRI), National Agricultural Research Centre (NARC), Islamabad 45500, Pakistan.

^2^ Department of Zoology, University of Science and Technology, Bannu, Pakistan.

^3^ Institute of Environmental Sciences and Engineering, School of Civil and Environmental Engineering, National University of Sciences and Technology, Islamabad, Pakistan.

^4^ School of Food and Agriculture, University of Management and Technology, Lahore 54000, Pakistan

^5^ College of Health Sciences, Abu Dhabi University, Abu Dhabi 59911, United Arab Emirates

^6^ Higher Education Commission, Islamabad, Pakistan

* Corresponding author:

**Iftikhar Ahmed**

ORCID: 0000-0003-0969-7927

E-mail : iftikhar.ahmed@parc.gov.pk

Tel: 92-51-9073 3177

**Table S1.** Identification of isolated heavy metal tolerant strains based on 16S rRNA gene sequence, their accession numbers published in DNA database and their NaCl tolerance.

| **Strain ID** | **Number of nucleotides of 16S rRNA gene** | **Accession number of 16S rRNA gene** | **Closely related validly published taxa** | **Similarity %age of 16S rRNA gene sequence with closely related species** | **Coverage** | **No. of closely related species having >97% (>98%) similarity of 16S rRNA gene sequence** | **NaCl Range (%)for growth (Optimum)** |
| --- | --- | --- | --- | --- | --- | --- | --- |
| NCCP-601 | 1416 | AB920786 | *Staphylococcus xylosus* (D83374) | 99.93 | 96.0 | 37 (21) | 0-18 (2) |
| NCCP-602 | 1432 | AB920787 | *Brevibacterium ammoniilyticum* (JF937067) | 98.56 | 97.0 | 12 (4) | 0-16 (2) |
| NCCP-603 | 1393 | AB920788 | *Acinetobacter guillouiae* (APOS01000028) | 98.63 | 95.4 | (2) | 0-4 (0) |
| NCCP-604 | 1417 | AB920789 | *Providencia vermicola* (AM040495) | 99.72 | 96.9 | 10 (9) | 0-10 (1) |
| NCCP-605 | 1393 | AB920790 | *Citrobacter youngae* (AJ564736) | 100.00 | 95.2 | 62 (29) | 0-8 (1) |
| NCCP-606 | 1393 | AB920791 | *Enterobacter aerogenes* (CP002824) | 99.43 | 95.3 | 62 (31) | 0-5 (1) |
| NCCP-607 | 1427 | AB920792 | *Enterobacter aerogenes* (CP0028240) | 99.02 | 96.6 | 57 (20) | 0-7 (1) |
| NCCP-608 | 1092 | AB920793 | *Enterobacter aerogenes* (CP002824) | 99.45 | 74.6 | 63 (30) | - |
| NCCP-609 | 1092 | AB920794 | *Citrobacter youngae* (AJ564736) | 100.00 | 74.5 | 59 (28) | 0-8 (1) |
| NCCP-610 | 832 | AB920795 | *Citrobacter youngae* (AJ564736) | 99.77 | 56.8 | 42 (8) | 0-8 (1) |
| NCCP-611 | 1432 | AB920796 | *Raoultella ornithinolytica* (AJ251467) | 99.72 | 98.0 | 53 (12) | 0-7 (1) |
| NCCP-612 | 984 | AB920797 | *Staphylococcus equorum* subsp. *equorum* (AB009939) | 100.00 | 66.7 | 22 (12) | 0-15 (2) |
| NCCP-613 | 1415 | AB920798 | *Citrobacter youngae* (AJ564736) | 97.94 | 95.2 | 1 | 0-8 (1) |
| NCCP-614 | 874 | AB920799 | *Stenotrophomonas rhizophila* (AJ293463) | 98.97 | 59.6 | 30 (2) | 0-4 (0) |
| NCCP-615 | 961 | AB920800 | *Alcaligenes aquatilis* (AJ937889) | 99.90 | 65.8 | (4) | 0-7 (1) |
| NCCP-616 | 1368 | AB920801 | *Alcaligenes aquatilis* (AJ937889) | 100.00 | 94.2 | (4) | - |
| NCCP-617 | 1356 | AB920802 | *Enterobacter aerogenes* (CP002824) | 98.60 | 93.0 | 40 (9) | - |
| NCCP-618 | 1376 | AB920803 | *Brevibacterium frigoritolerans* (AM747813) | 99.93 | 93.5 | 7 (4) | 0-6 (1) |
| NCCP-619 | 1241 | AB920804 | *Bacillus persicus* (HQ433471) | 99.52 | 66.2 | 27 (1) | 0-9 (1) |
| NCCP-620 | 1087 | AB920805 | *Klebsiella pneumoniae subsp. rhinoscleromatis* (ACZD01000038) | 99.63 | 74.4 | 47 (20) | 0-10 (1) |
| NCCP-621 | 852 | AB920806 | *Exiguobacterium indicum* (AJ846291) | 100.00 | 57.4 | 8 (5) | 0-9 (1) |
| NCCP-622 | 1399 | AB920807 | *Brevibacterium frigoritolerans* (AM747813) | 100.00 | 95.0 | 7 (4) | 0-6 (1) |
| NCCP-623 | 815 | AB920808 | *Klebsiella pneumoniae* subsp. *pneumoniae* (AJJI01000018) | 99.75 | 55.7 | 60 (24) | - |
| NCCP-624 | 970 | AB920809 | *Pseudomonas veronii* (AF064460) | 99.59 | 66.4 | 49 (32) | 0-4 (0) |
| NCCP-628 | 1132 | AB920810 | *Staphylococcus equorum subsp. equorum* (AB009939) | 100.00 | 76.8 | 24 (12) | 0-15 (2) |
| NCCP-630 | 1023 | AB920811 | *Pseudomonas aeruginosa* (Z76651) | 99.90 | 70.1 | (2) | 0-4 (0) |
| NCCP-631 | 861 | AB920812 | *Klebsiella pneumoniae* subsp. *rhinoscleromatis* (ACZD01000038) | 99.77 | 58.8 | 58 (28) | - |
| NCCP-632 | 946 | AB920813 | *Pseudomonas aeruginosa* (Z76651) | 99.89 | 64.8 | 3 (2) | - |
| NCCP-633 | 1049 | AB920814 | *Pseudomonas aeruginosa* (Z76651) | 99.81 | 72.0 | (2) | - |
| NCCP-634 | 1133 | AB920815 | *Staphylococcus equorum* subsp*. equorum* (AB009939) | 99.82 | 76.8 | 20 (11) | - |
| NCCP-635 | 1099 | AB920816 | *Paenibacillus motobuensis* (AY741810) | 99.72 | 74.5 | (1) | 0-5 (0) |
| NCCP-636 | 1155 | AB920817 | *Planococcus rifietoensis* (AJ493659) | 99.83 | 78.4 | 19 (10) | - |
| NCCP-637 | 1266 | AB920818 | *Bacillus flexus* (AB021185) | 99.37 | 86.3 | 4 (4) | - |
| NCCP-638 | 965 | AB920819 | *Bacillus flexus* (AB021185) | 100.00 | 65.4 | 4 (4) | - |
| NCCP-640 | 976 | AB920820 | *Pseudomonas aeruginosa* (Z76651) | 99.90 | 67.0 | 2 (2) | - |
| NCCP-642 | 993 | AB920821 | *Bacillus anthracis* (AB190217) | 100.00 | 67.4 | 12 (10) | - |
| NCCP-643 | 950 | AB920822 | *Bacillus anthracis* (AB190217) | 100.00 | 64.5 | 12 (10) | - |
| NCCP-644 | 1413 | AB916465 | *Acinetobacter kyonggiensis* (FJ527818) | 98.33 | 96.7 | 10 (1) | 0-4 (0) |
| NCCP-645 | 966 | AB920823 | *Pseudomonas fragi* (AF094733) | 99.38 | 66.3 | 50 (22) | 0-5 (0) |
| NCCP-646 | 840 | AB920824 | *Pseudomonas azotoformans* (D84009) | 98.20 | 57.5 | 9 (1) | 0-5 (1) |
| NCCP-647 | 1124 | AB920825 | *Staphylococcus equorum* subsp. *equorum* (AB009939) | 99.73 | 75.9 | 18 (10) | 0-8 (1) |
| NCCP-648 | 1390 | AB920826 | *Psychrobacter faecalis* (AJ421528) | 99.78 | 95.1 | 27 (9) | - |
| NCCP-649 | 532 | AB920827 | *Stenotrophomonas rhizophila* (AJ293463) | 99.25 | 36.2 | 4 (1) | - |
| NCCP-650 | 1412 | AB920828 | *Alcaligenes faecalis* subsp. *parafaecalis* (AJ242986) | 98.78 | 96.5 | 4 (3) | 0-7 (1) |
| NCCP-651 | 1403 | AB920829 | *Bacillus cereus* (AE016877) | 100.00 | 95.1 | 12 (11) | 0-8 (1) |
| NCCP-652 | 846 | AB920830 | *Thauera mechernichensis* (Y17590) | 99.05 | 58.0 | 6 (6) | 0-4 (0) |
| NCCP-653 | 1134 | AB920831 | *Bacillus aerophilus* (AJ831844) | 99.47 | 76.6 | 5 (5) | - |
| NCCP-654 | 1118 | AB920832 | *Pseudomonas extremaustralis* (AHIP01000073) | 99.46 | 76.3 | 48 (30) | - |
| NCCP-655 | 1119 | AB920833 | *Stenotrophomonas rhizophila* (AJ293463) | 99.46 | 76.2 | 6 (2) | 0-8 (1) |
| NCCP-656 | 1091 | AB920834 | *Bacillus safensis* (AF234854) | 99.54 | 74.1 | 5 (5) | - |
| NCCP-657 | 1401 | AB920835 | *Bacillus subtilis* subsp. *inaquosorum* (AMXN01000021) | 99.36 | 95.1 | 15 (12) | - |
| NCCP-658 | 1407 | AB920836 | *Bacillus gibsonii* (X76446) | 99.64 | 95.2 | 3 (3) | - |
| NCCP-659 | 1400 | AB920837 | *Bacillus safensis* (AF234854) | 99.93 | 95.0 | 10 (5) | - |
| NCCP-660 | 881 | AB920838 | *Staphylococcus haemolyticus* (L37600) | 100.00 | 59.7 | 38 (16) | 0-20 (2) |
| NCCP-661 | 1092 | AB920839 | *Brachybacterium nesterenkovii* (X91033 ) | 99.45 | 75.6 | 6 (2) | 0-9 (1) |
| NCCP-662 | 1492 | AB968093 | *Bacillus niabensis* (AY998119) | 98.17 | 100 | 3 (2) | 0-10 (1) |
| NCCP-663 | 858 | AB968094 | *Alcaligenes faecalis subsp. faecalis* (D88008) | 99.18 | 58.9 | 4 (4) | 0-9 (1) |
| NCCP-666 | 1494 | AB968095 | *Bacillus cohnii* (X76437) | 99.86 | 100 | 4 (2) | 0-9 (1) |
| NCCP-667 | 1479 | AB968096 | *Alcaligenes aquatilis* (JX986974) | 99.32 | 100 | 4 (3) | 0-7 (1) |
| NCCP-668 | 829 | AB968097 | *Citrobacter amalonaticus* (FR870441) | 100.00 | 56.6 | 24 (10) | 0-10 (1) |
| NCCP-670 | 811 | AB968098 | *Brevundimonas mediterranea* (AJ227801) | 99.88 | 58.6 | 8 (5) | 0-4 (0) |
| NCCP-671 | 882 | AB968099 | *Bacillus sonorensis* (AYTN01000016) | 99.21 | 59.9 | 15 (10) | 0-20 (2) |
| NCCP-672 | 789 | AB968100 | *Staphylococcus xylosus* ([D83374](http://www.ezbiocloud.net/eztaxon/hierarchy?m=browse&k=D83374&d=2&s=search)) | 99.87 | 53.5 | 23 (12) | 0-20 (2) |
| NCCP-673 | 892 | AB968101 | *Exiguobacterium mexicanum* (AM072764) | 100.00 | 60.1 | 8 (6) | 0-12 (1) |
| NCCP-675 | 868 | AB968102 | *Raoultella ornithinolytica* (AJ251467) | 100.00 | 59.4 | 55 (21) | 0-7 (1) |
| NCCP-677 | 1492 | AB968103 | *Paenibacillus motobuensis* (AY741810) | 99.86 | 100 | 1 (1) | 0-4 (0) |
| NCCP-679 | 828 | AB968104 | *Enterobacter aerogenes* (CP002824) | 99.28 | 56.7 | 55 (33) | 0-7 (1) |
| NCCP-680 | 1501 | AB968105 | *Acinetobacter bouvetii* (APQD01000004) | 99.93 | 100 | 12 (5) | 0-3 (0) |

**Table S2. Set of primers (used in present study), annealing temperature and expected amplicon size.**

| Target genes | Primer pair for amplification | Sequence | Annealing temp. (°C) | Expected amplicon size of target gene (bp) | Reference |
| --- | --- | --- | --- | --- | --- |
| *nifH* | PolF (forward) | 5' - TGC GAY CCS AAR GCB GAC TC - 3' | 50 °C | 360 | ([Poly et al. 2001](#_ENREF_3)) |
|  | PolR (reverse) | 5' - ATS GCC ATC ATY TCR CCG GA - 3' |  |  |  |
| *nifH* | nifHF (forward) | 5' -TACGGNAARGGSGGNATCGGCAA - 3' | 57 °C | 780 | ([Laguerre et al. 2001](#_ENREF_2)) |
|  | nifHI (reverse) | 5' – AGCATGTCYTCSAGYTCNTCCA - 3' |  |  |  |
| *nifH* | nifHfor (forward) | 5' - TAYGGNAARGGNGGHATYGGYATC - 3' | 50 °C | 420 | ([Sarita et al. 2008](#_ENREF_4)) |
|  | nifHrev (reverse) | 5' - ATRTTRTTNGCNGCRTAVABBGCCATCAT - 3' |  |  |  |
| *acdS* | F1936 (forward) | 5'- GH GAM GAC TGC AAY WSY GGC - 3' | 50 °C | 792 | ([Blaha et al. 2006](#_ENREF_1)) |
|  | F1938 (reverse) | 5'- AT CAT VCC VTG CAT BGA YTT - 3' |  |  |  |
| *acdS* | F1936 (forward) | 5'- GH GAM GAC TGC AAY WSY GGC - 3' | 50 °C | 558 |  |
|  | F1939 (reverse) | 5' - GA RGC RTC GAY VCC RAT CAC - 3' |  |  |  |
| *acdS* | F1937 (forward) | 5' - MGV AAG CTC GAA TAY MTB RT - 3' | 50 °C | 516 |  |
|  | F1939 (reverse) | 5' - GA RGC RTC GAY VCC RAT CAC - 3' |  |  |  |

**References**

Blaha D, Prigent‐Combaret C, Mirza MS, Moënne‐Loccoz Y (2006) Phylogeny of the 1‐aminocyclopropane‐1‐carboxylic acid deaminase‐encoding gene *acdS* in phytobeneficial and pathogenic Proteobacteria and relation with strain biogeography FEMS microbiology ecology 56:455-470

Laguerre G, Nour SM, Macheret V, Sanjuan J, Drouin P, Amarger N (2001) Classification of rhizobia based on *nodC* and *nifH* gene analysis reveals a close phylogenetic relationship among *Phaseolus vulgaris* symbionts Microbiol 147:981-993

Poly F, JocteurMonrozier L, Bally R (2001) Improvement in the RFLP procedure for studying the diversity of nifH genes in communities of nitrogen fixers in soil Res Microbiol 152 95–103

Sarita S, Priefer UB, Prell J, Sharma PK (2008) Diversity of *nifH* gene amplified from rhizosphere soil DNA Curr Sci 94:109-115

**Table S3:** *nifH* and *acdS* genes amplification with different sets of primers.

| **Name of Strain** | ***nif*H gene**  (PolF / PolR) | ***nif*H gene**  (nifHF / nifHI) | ***nif*H gene**  (nifHfor / nifHrev) | ***acdS* gene**  (F1936f / F1938r) | ***acdS* gene**  (F1936 / F1939r) | ***acdS* gene**  (F1937f / F1939r) |
| --- | --- | --- | --- | --- | --- | --- |
| NCCP-601 | – | –w | –w | –w | –w | –w |
| NCCP-602 | –w | –w | –w | –w | –w | –w |
| NCCP-605 | –w | –w | + | –w | +m | –w |
| NCCP-606 | –w | ND | + | –w | –w | –w |
| NCCP-607 | +m | ND | +m | –w | –w | –w |
| NCCP-611 | +m | –w | + | –w | +m | +m |
| NCCP-614 | –w | –w | +m | –w | –w | +m |
| NCCP-621 | –w | +m | + | –w | –w | –w |
| NCCP-622 | + | –w | –w | +m | –w | –w |
| NCCP-624 | –w | –w | – | –w | –w | –w |
| NCCP-625 | + | +m | –w | –w | –w | –w |
| NCCP-635 | +m | ND | –w | +m | –w | –w |
| NCCP-644 | –w | –w | –w | –w | –w | –w |
| NCCP-645 | –w | –w | – | – | –w | –w |
| NCCP-646 | –w | ND | –w | +m | –w | –w |
| NCCP-647 | –w | –w | – | –w | –w | –w |
| NCCP-650 | +m | +m | – | +m | +m | +m |
| NCCP-651 | – | –w | – | –w | –w | –w |
| NCCP-652 | –w | –w | –w | –w | –w | –w |
| NCCP-655 | ND | +m | + | –w | –w | – |
| NCCP-660 | **+** | ND | ND | – | –w | +m |
| NCCP-661 | +m | –w | – | – | –w | –w |
| JCM 10833 | + | –w | –w | +m | –w | –w |
| JCM 21823 | + | –w | –w | –w | –w | –w |

ND, not determined; +, PCR product of the expected size; –, no PCR product; -w, no expected PCR products, only products of unexpected and nonspecific size; +m, expected PCR products plus other products of unexpected and nonspecific size.
